# Generation of the First Structure-Based Pharmacophore Model Containing a Selective “Zinc Binding Group” Feature to Identify Potential Glyoxalase-1 Inhibitors

**DOI:** 10.3390/molecules171213740

**Published:** 2012-11-22

**Authors:** Qosay Al-Balas, Mohammad Hassan, Buthina Al-Oudat, Hassan Alzoubi, Nizar Mhaidat, Ammar Almaaytah

**Affiliations:** 1 Department of Medicinal Chemistry and Pharmacognosy, Faculty of Pharmacy, Jordan University of Science and Technology, P.O. Box 3030, Irbid 22110, Jordan; E-Mails: hassan-m@just.edu.jo (M.H.); baoudat@just.edu.jo (B.A.-O.); haszoubi@just.edu.jo (H.A.); 2 Department of Clinical Pharmacy, Faculty of Pharmacy, Jordan University of Science and Technology, P.O. Box 3030, Irbid 22110, Jordan; E-Mail: nizarm@just.edu.jo; 3 Department of Pharmaceu tical Technology, Faculty of Pharmacy, Jordan University of Science and Technology, P.O. Box 3030, Irbid 22110, Jordan; E-Mail: amalmaaytah@just.edu.jo

**Keywords:** glyoxlase-1 enzyme, zinc binding group, GOLD, CDOCKER, molecular docking, 2D similarity search

## Abstract

Within this study, a unique 3D structure-based pharmacophore model of the enzyme glyoxalase-1 (Glo-1) has been revealed. Glo-1 is considered a zinc metalloenzyme in which the inhibitor binding with zinc atom at the active site is crucial. To our knowledge, this is the first pharmacophore model that has a selective feature for a “zinc binding group” which has been customized within the structure-based pharmacophore model of Glo-1 to extract ligands that possess functional groups able to bind zinc atom solely from database screening. In addition, an extensive 2D similarity search using three diverse similarity techniques (Tanimoto, Dice, Cosine) has been performed over the commercially available “Zinc Clean Drug-Like Database” that contains around 10 million compounds to help find suitable inhibitors for this enzyme based on known inhibitors from the literature. The resultant hits were mapped over the structure based pharmacophore and the successful hits were further docked using three docking programs with different pose fitting and scoring techniques (GOLD, LibDock, CDOCKER). Nine candidates were suggested to be novel Glo-1 inhibitors containing the “zinc binding group” with the highest consensus scoring from docking.

## 1. Introduction

Cancer is defined as a renegade system of growth that originates in human body. It is characterized, regardless of its type, by one common feature, which is unchecked growth that progresses toward limitless cell expansion. This process of unlimited proliferation is due to the fact that cells have lost their ability to undergo programmed suicide based on a process called apoptosis [1]. According to the World Health Organization [WHO] statistics, cancer is considered the leading cause of death worldwide, responsible for 7.6 million deaths in 2008. Lung, stomach, liver, colon and breast cancers cause the highest numbers of cancer deaths each year, distributed between males and females. The rate of cancer incidence continues to increase, with around 13.0 million cases expected in 2030. In addition, it is expected that more than 70% of the casualties are from medium and low income countries [2,3].

For many years, there have been continuous efforts by the scientists to find new valid targets for cancer treatment and to discover new chemotherapeutic agents; consequently, their efforts have contributed to a decline in the mortality rate, but until today, there is still no chemotherapeutic agent that possesses the optimal characteristics for cancer treatment due to the lack of selectivity between normal cells and cancer ones. Based on this fact, there is a continuous search to find candidate drugs that provide the best treatment and medical relief for suffering patients [4,5].

One of the valid targets acknowledged to be pivotal for cancer treatment is the glyoxalase system enzymes. This system is composed of two enzymes, glyoxalase-1 (Glo1) and glyoxalase-2 (Glo2). These enzymes are mainly involved in the detoxification mechanism of metabolic byproducts, especially the reactive aldehydes such as methylglyoxal (MG), that are produced inside the cells during the normal process of metabolism. Glo1 is the first enzyme in the series that is responsible for the isomerization process of the toxic metabolite (MG), followed by Glo2 that performs the hydrolysis step to produce innocent bystanders that are safely excreted outside the body [6,7,8,9]. The glyoxalase system has been proven to be found in both normal and malignant cells, however, with much higher rate expression in tumor cells. Moreover, it has been emphasized that this over-expression confers resistance to anti-tumor agents such as adriamycin and etoposide [10,11,12,13].

**Scheme 1:**
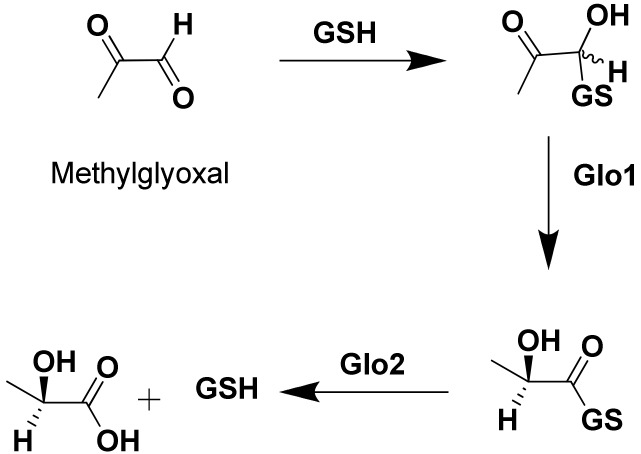
Mechanism of methylglyoxal detoxification utilizing the glyoxalase system enzymes.

Based on Scheme 1 scientists have proposed that inhibition of either Glo1 or Glo2 will result in an accumulation of toxic and reactive aldehydes, which can lead to self-destruction of cancer cells. Cancer cells are characterized by being very active metabolically; they do not stop growing and usually consume abundant amounts of nutrients. Therefore, inhibition of Glo1 and/or Glo2 should result in production of toxic metabolites including aldehydes MG inside these malignant cells, which can lead to the death of cancer cells due to toxins accumulation [14,15,16].

Glo1 has received particular attention in the last decade as an anti-cancer target with many publications assuring its importance as an anti-cancer valid target. Structurally, Glo1 is a metallo-enzyme, *i.e.*, it has zinc metal as an essential cofactor inside the active site, which plays a structural and catalytic role in the isomerization of the reactive and toxic aldehydes. In addition, it is composed of two identical polypeptide chains forming a homodimer, where each chain is composed of 183 amino acid with a molecular weight of 43 kDa. The active site of Glo1 is situated in the interface of the two polypeptide chains and the zinc atom has a square pyramidal coordination with the adjacent amino acids [17,18].

**Figure 1:**
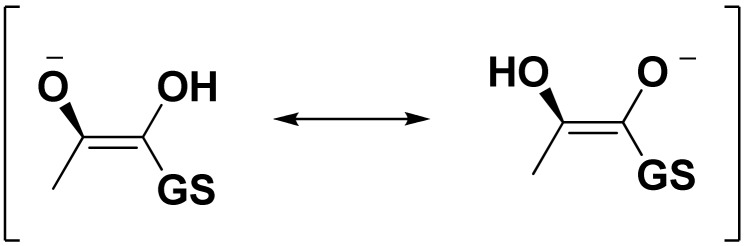
Transition state inside the active site of Glo1.

Designing competitive inhibitors for Glo1 has been performed mainly by three research groups. First, More *et al.* have designed competitive inhibitors based on the transition state of MG inside the active side (Figure 1), so they build their inhibitors based on S-*p*-bromobenzyl glutathione which was discovered to be a valid inhibitor [19].

**Figure 2:**
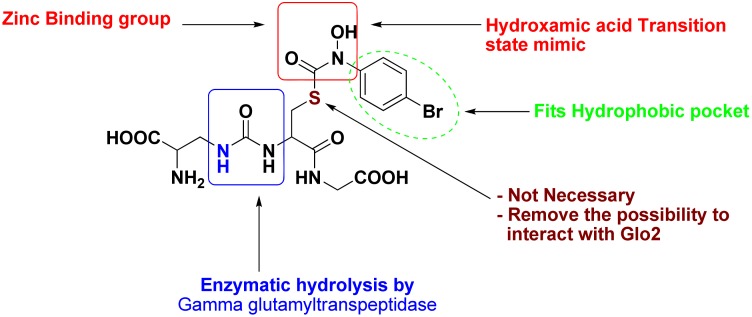
More *et al.* structural modifications to synthesize transition state competitive inhibitors of Glo1.

The strategy adopted by More *et al.* was to design an inhibitor that has zinc binding group and resist hydrolysis by peptidases especially Gamma glutamyltranspeptidase (Figure 2).

Hydroxamic acid was chosen as a zinc binding group which also resembles the transition state. This group was found to be unstable due to its feature of being a good leaving group. Therefore, a retro- synthesis was performed to reverse the orientation of hydroxamic acid and this produced a stable zinc binding group. The other strategy was to protect the compound from enzymatic hydrolysis, so an amide bond was isosterically replaced by an ureido derivative, which is more resistant to hydrolysis. Moreover, for simplification purposes, his group has emphasized that the sulfur atom is not necessary for pharmacodynamic purposes. It was replaced by a carbon atom to facilitate synthesis of new derivatives. Due to the difficulties that involved in the hydroxamic acid synthesis, More *et al*. shifted the synthesis from hydroxamic acid derivatives to acetoacetate transition state inhibitors. This shift was required to enrich the diversity of the synthesized compounds to find a suitable inhibitor with an acceptable activity [20,21,22,23,24].

**Figure 3:**
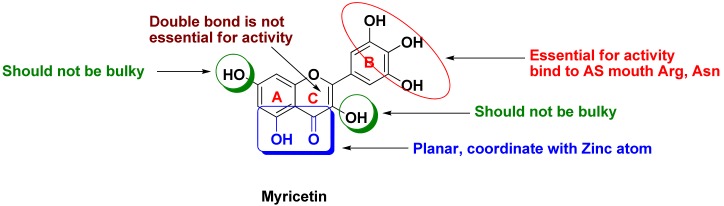
Takasawa vision of the expected pharmacophore and the SAR of flavonoids binding to Glo1.

**Figure 4:**
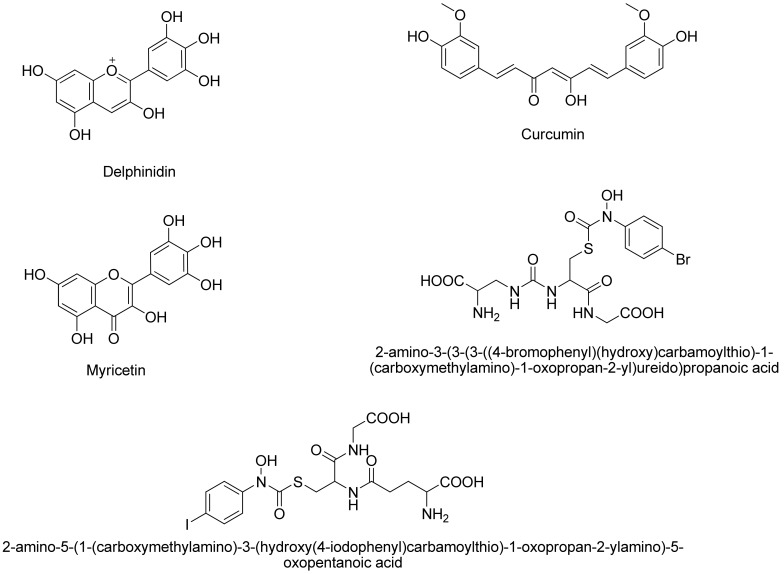
Structure of the five compounds reported as inhibitors of Glo-1 enzyme.

Takasawa *et al*., on the other hand, have exploited the presence of the planar C-4 ketone and C-5 hydroxy groups found in natural flavonoids to test them as Glo1 binders (Figure 3). Luckily, he discovered that these compounds possess the needed structure to chelate the zinc atom inside the active site. Further studies performed by his group have proved that the polyhydroxyl groups on the B ring found in the flavonoid structure plays an important role in binding to the amino acids at the mouth of the active site such as Asn and Arg. He described the structure activity relationship in this flavonoid scaffold by assuring that there should be a hydroxyl and ketone groups that are planar to each other and are separated by 2.5 ± 0.5Å while they are not surrounded by bulky substituents. Another natural compound belong to the anthocyanidines (Figure 4, delphinidin) has been discovered to bind to Glo1 and act as competitive inhibitor. The anthocyanidines’ positive charge undergoes *ionic-pi* interactions with aromatic amino acids inside the active site in addition to the OH interaction with the zinc atom. Like the flavonoids, the terminal hydroxyl groups are hydrogen bound with basic amino acids at the mouth of the active site [25,26,27]. 

A third major study conducted by Yuan *et al.* has investigated the binding mode of the natural substrate curcumin (Figure 4) by using molecular modeling and molecular dynamics. Their results showed that the enol form of curcumin possessed zinc binding properties and was much more stable than the keto form of the same compound [28,29]. 

In the present study, a series of potential Glo1 inhibitors are suggested based on structure-based pharmacophore design which has been conducted using Discovery Studio 3.1 (DS 3.1) from Accelrys to extract a 3D pharmacophore that reflects the important functional groups that are essential for inhibitor binding. In addition, a 2D similarity search has been employed based on known active inhibitors of Glo1 previously reported by literature to mine commercial databases for similar drug-like molecules. Finally, molecular docking studies were performed relying on three different programs employing different procedures for their docking and scoring.

## 2. Results and Discussion

### 2.1. Structure-Based Pharmacophore Generation

**Figure 5:**
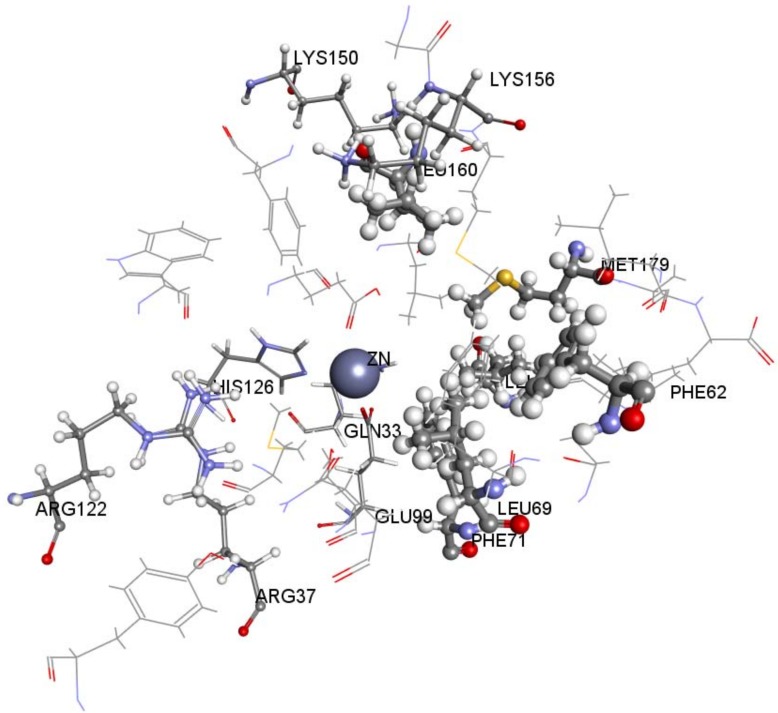
A 3D view representing the three main areas that forms the active site of Glo-1 enzyme. Zn atom is represented by CPK and the hydrophobic pocket and the active site mouth residues are represented by scaled balls and sticks.

A *Receptor-Ligand Pharmacophore Generation* protocol produced ten pharmacophore models ranking them according to their selectivity score, the higher the better. According to the results, ten pharmacophores that scored selectivity scores from 8.54 to 7.59 were generated from eleven features that matched the receptor-ligand interactions HBA,HBA,HBA,HBA,HBD,HBD,HBD,HY,HY,NI,NI (Table 1). These interactions revealed the important amino acids that were later used to help decide the final structure-based pharmacophore model. The active site of Glo-1 enzyme can be described as having three major binding areas (Figure 5). The first and the most important area is the zinc atom; it is positioned at the bottom of the active site and forms coordinate bonds with three amino acids: Gln33, His126 and Glu99. The zinc atom is able to form an extra coordinate bond with the zinc binding group provided by the inhibitor and this justifies the essentiality of the presence of a zinc binding group when the inhibitor is being designed. Secondly, a small hydrophobic pocket that is inserted deeply inside the active site that can tolerate up to two aromatic rings can be exploited. This hydrophobic area is formed from the following amino acids; Leu92, Phe71, Met179, Leu160, Leu69 and Phe62. Finally, the mouth of the active site, which is composed of Arg122, Arg37, Lys150 and Lys156, is highly polar. All ten of the pharmacophore models generated from the bound ligand to 1QIN considered the zinc atom as HBD (which later will be customized to be the zinc binder feature). The hydrophobic pocket was filled by one or two HY features in all ten generated pharmacophores. On the other hand, the mouth of the active site was represented by HBD, HBA and NI features correlated to the polar amino acids that are present there. 

The *Interaction Generation* protocol has also been used in building the structure-based pharmacophore model. However, this protocol differs from the previous one in that it maps the polar and apolar areas in the active site regardless of any ligands bound to the active site. The results have shown a similar trend to the *Receptor-Ligand Pharmacophore Generation* protocol by indicating the presence of a hydrophobic pocket, and the HBD feature points to the zinc atom and polar areas in the mouth of the active site, so by combining the results of the two pharmacophores, a final pharmacophore model has been achieved that satisfies the essential features inside the active site.

**Figure 6:**
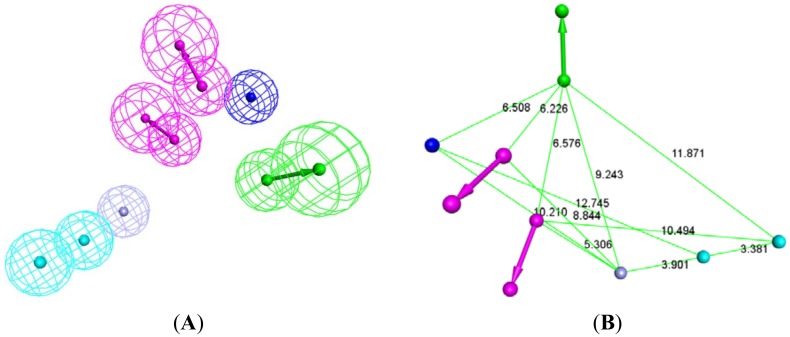
Structure-based pharmacophore of the active site of Glo-1 enzyme. (**A**) 3D representation of this pharmacophore; HY: sky-blue; NI: blue; HBD: magenta; HBA: green; ZB: purple. (**B**) simplified structure-based pharmacophore with no tolerance spheres to facilitate visualization of inter feature distances; HY: sky-blue; NI: blue; HBD: purple; HBA: green; ZB: purple.

The produced pharmacophore model has been customized in order to add a zinc binding feature inside the active site instead of HBD, so the coordinates of zinc atom inside the active site were assigned and the zinc feature was added (Figure 6). This new pharmacophore model contains seven features; ZB points to zinc atom, 2HY features filling the hydrophobic pocket, 2HBD pointing to amino acids near the active site mouth Asn103 and Thr101, HBA points to Lys156 and NI that is complimentary to Arg122 and Arg37. The final generated pharmacophore will be used later to help filter drug candidates from 2D similarity search. 

### 2.2. 2D Similarity Search

Within this work, a 2D similarity search was used to virtually screen a large database called Zinc-Clean Drug-Like Database containing 9,542,593 compounds based on five active compounds reported as active inhibitors of Glo-1. The five compounds shown in Figure 4 were selected based on the fact that compound 2-amino-5-(1-(carboxymethylamino)-3-(hydroxy(4-iodophenyl)carbamoylthio)-1-oxopropan-2-ylamino)-5-oxopentanoic acid is co-crystallized inside the active site of Glo-1 enzyme as an inhibitor; consequently, the compound 2-amino-3-(3-(3-((4-bromophenyl)(hydroxy)carbamoylthio)-1-(carboxymethylamino)-1-oxopropan-2-yl)ureido)propanoic acid was suggested as potential inhibitor by More *et al.* based on transition state inhibitor design. Curcumin, myricetin and delphinin have been reported to be potential inhibitors for Glo1 enzyme and contain zinc binding scaffolds [25,26,27]. 

**Figure 7:**
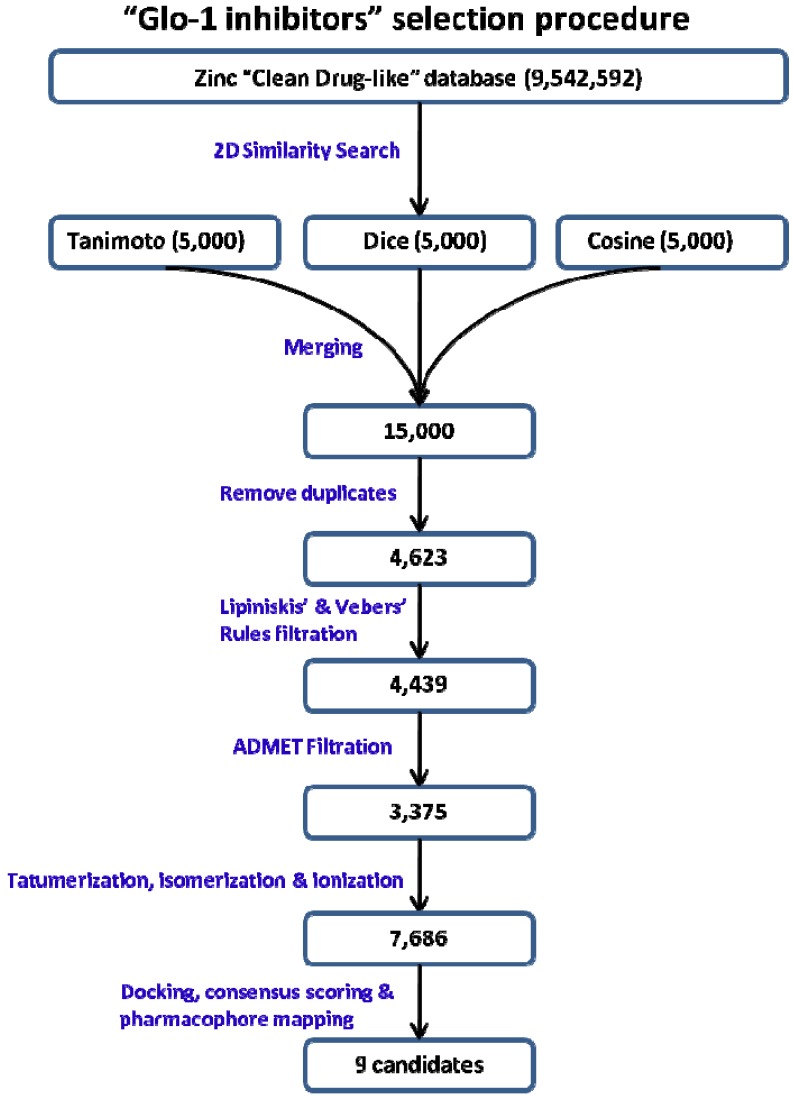
Steps in the drug discovery process to find potential inhibitors of Glo-1 enzyme.

2D similarity virtual screening techniques have been extensively used to find similar molecules to known inhibitors based on the idea that “molecules that are structurally similar are likely to have similar properties”, first enunciated explicitly by Johnson and Maggoria [30,31,32,33]. Each screening run of this database will produce the 500 molecules most similar to the queried active structures using 2D similarity measures (Tanimoto, Dice, and Cosine) and each measure will use two fingerprints (FCFP_6 and ECFC_6). Using three measures will add credibility to the produced results as each similarity measure applies different equation with different parameters; so, a total of 1,500 returned similar compounds can be obtained by applying the three measures for each queried compound. In addition, each measure uses two fingerprints which also adds further credibility to the results. FCFP_6 searches for similarity that is based on the functional class such as HBD, HBA, and RA properties that are shared between the queried reference molecule and the target molecules in the database. On the other hand, ECFC-6 searches for similarity based on atom types such as hybridization and charge. Combining both methodologies will produce diverse results and expand the similarity to its maximum as the number 6 at the end of these fingerprints is indicative to the maximum range searched by the algorithm. Consequently, each searched molecule will return 3,000 molecules similar to it. Since five molecules needed to be queried, the total compounds returned by this research is 15,000 molecules (Figure 7) The 15,000 compounds are further filtered in order to narrow the possible candidates starting by merging these compounds in one *sd* file, as each run returns 500 molecules. The next step is to remove duplicate structures that are produced from these runs by applying the *Prepare Ligands* protocol and activate remove duplicate option only,; this step reduces the candidate structures to 4,623. Furthermore, the resulting structures are reduced to 4,439 by applying *Filter by Lipinski and Veber Rules* protocol which filters molecules according to their drug likeness and their suitability to be orally bioavailable drug candidates. These rules limit the structures to be good orally active to have five or less HBDs, 10 or less HBA, a logP value of 5 or less, molecular weight less than 500, rotatable bonds 10 or less and polar surface area not exceeding 140Å^2^. More filtration steps were conducted by applying ADMET rules, so the candidates will be more drug-like structures within this protocol. Structures were filtered according to their solubility, intestinal absorption and Blood Brain Barrier penetration and 3,375 passed this step. The following step resulted in an increase of the candidate structures rather than a reduction of their number. This is related to preparing the filtered compounds for 3D structure-based pharmacophore screening and molecular docking, so the 3,375 structures were approximately doubled to 7,686 structures after applying the *Prepare Ligands* protocol which performs tautomerization, ionization to pH range 6.5–8.5 and creates isomers of the selected structures. This step is essential because it expresses all the expected chemical forms that the structure could form while it is inside the body at physiological conditions. Moreover, it has been reported that tautomerization is an essential step in the process of virtual docking of compounds containing zinc atoms in the active site [34,35,36]. 

### 2.3. Mapping 2D Similarity Search Compounds over Structure-Based Pharmacophore

**Figure 8:**
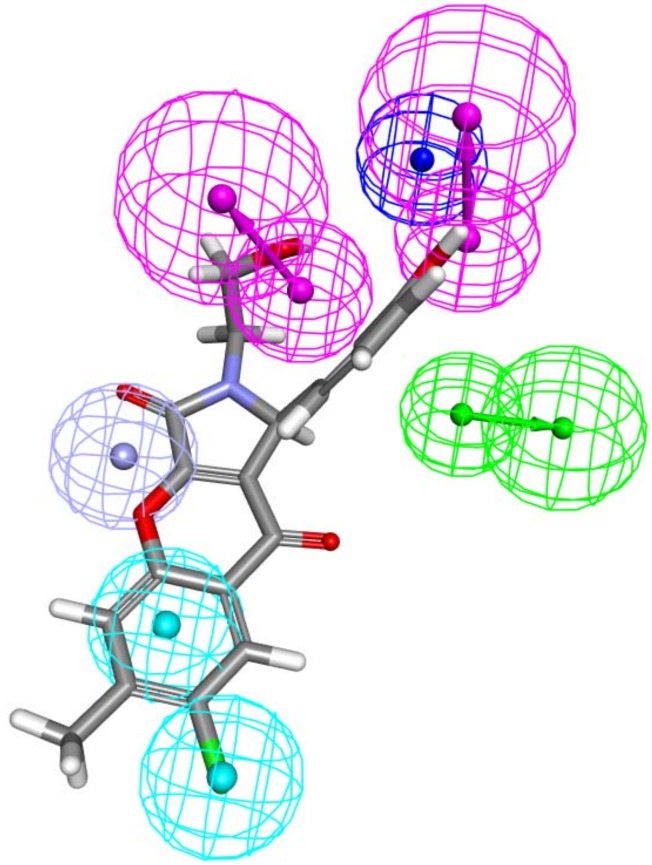
Zinc02230638 mapped over the generated structure-based pharmacophore.

At this step, it is important to find candidate structures that share similarity with active molecules reported in the literature and fulfill the requirements of being able to fit in the generated structure-based pharmacophore model which is a reflection of the active site geometry. Consequently, both 2D and 3D criteria will be attained. The *Screen Library* protocol was run for the 7,686 compounds generated from 2D similarity search by applying *Best Conformation* generation of 255 conformers for each structure with 20 kcal energy threshold. This screening step has returned 2,373 compounds able to match the structure-based pharmacophore with a minimum of four features out of seven being satisfied, and every compound should map the zinc binding feature and the HY feature adjacent to it. Compound zinc02230638 mapped to structure-based pharmacophore is depicted in Figure 8. Five features were fulfilled; the 1-pyrrol-2-one ring fits the ZB, the two HY features were mapped with chlorobenzene ring, and each of the two hydroxyl groups mapped to the two HBD features. The fit value for this compound was 4.3, while the other two features were not mapped- the HBA and NI. For the final selection of candidate inhibitors of Glo-1 enzyme, the fit values of these compounds will be compared with the consensus scoring of the molecular docking step.

### 2.4. Molecular Docking

**Figure 9:**
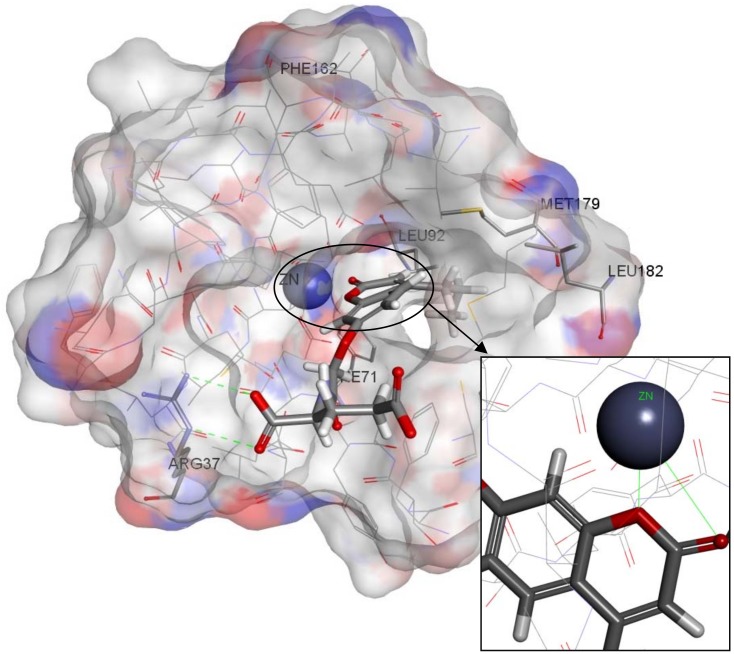
Zinc02120846 docked inside the Glo-1 active site.

**Figure 10:**
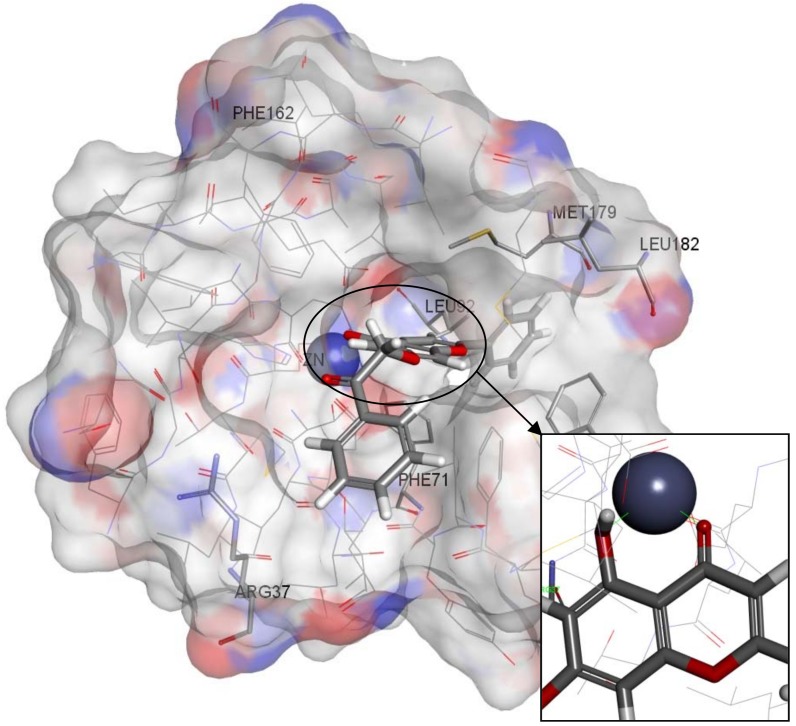
Zinc05528245 docked inside the Glo-1 active site.

**Figure 11:**
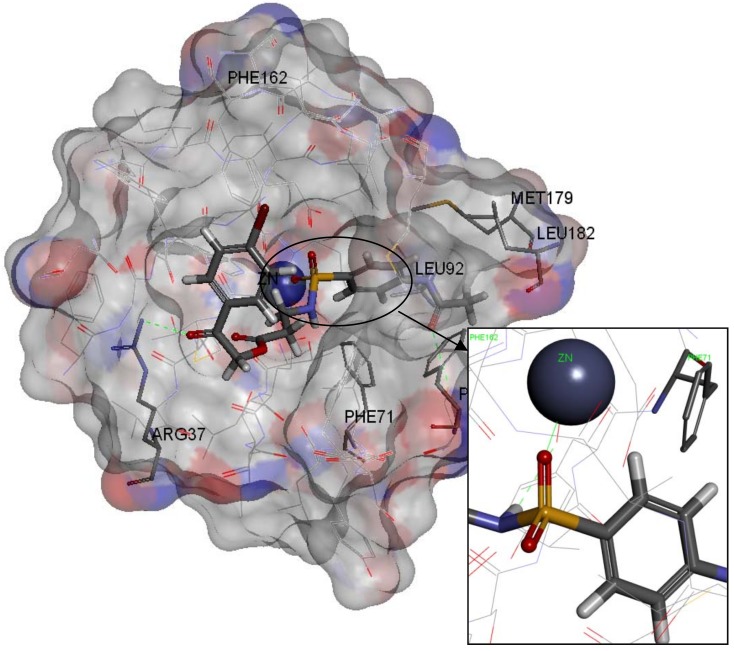
Zinc13100715 docked inside the Glo-1 active site.

**Figure 12:**
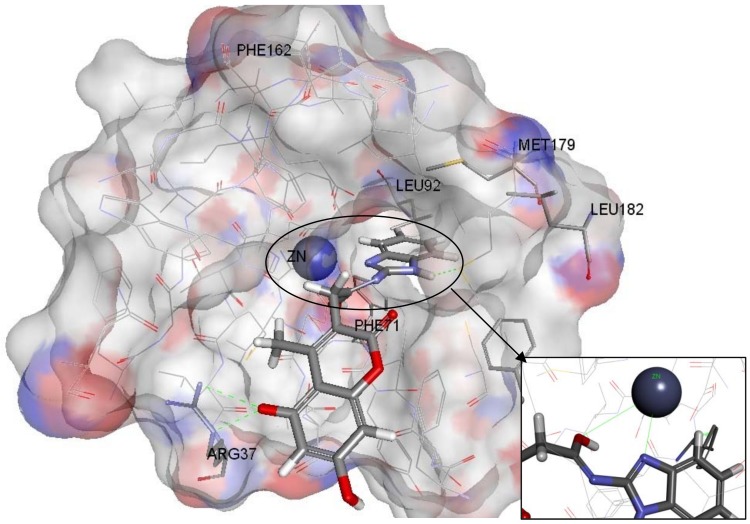
Zinc08791976 docked inside the Glo-1 active site.

The 7,686 compounds were next docked by using three docking programs with six scoring functions. Consensus scoring was performed by summing the values of the six scoring functions and then comparing them with the fitting values performed in the previous step. The candidate molecules were selected based on the ability of the chemical structures to map the generated structure-based pharmacophore, getting the highest ranks in the consensus scoring tactic and visually examining the binding pattern of the docked poses. Consequently, nine compounds were chosen to be potential inhibitors which contain various functional groups performing binding with zinc atom at the active site (Table 2). For example, Zinc02120846 is a 2*H*-chromen-2-one based structure in which the 2*H*-pyran-2-one ring coordinates with the zinc atom by the two oxygen atoms; the ketone oxygen with a 2.36 Å apart and the oxygen ring with 2.18 Å. One of the carboxylic acid groups performs two hydrogen bonds with Arg37 amino acid at the mouth of the active site. The lipophilic pocket of the active site is filled with n-propyl group performing hydrophobic interactions (Figure 9). Zinc05528245 possesses a 4-oxo-4*H*-chromen-5-olate central ring which plays a crucial role in chelating zinc atom with enol and ketone oxygens distances 2.43 Å and 2.12 Å, respectively. The benzene ring attached to the ketone points out to the mouth of the active site while the other benzene ring fills the hydrophobic pocket (Figure 10). Zinc13100715 is sulfonamide-based structure which has a chelating zinc atom at the active site, the bromobenzene ring points out to the opening of the active site while the adjacent ketone forms H-bonding with Arg37. The hydrophobic pocket is occupied by phenyacetamide group (Figure 11). Finally, Zinc08791976 is a benzimidazole-based structure in which the imidazole ring coordinated with zinc atom at the pyridine-like nitrogen, whereas the pyrrole-like nitrogen forms a H-bond with the sulfur atom of Met157. The benzene ring attached to the imidazole ring fills the hydrophobic pocket whereas the hydroxy-2-oxo-2*H*-chromen-5-olate ring points out to the mouth of the active site (Figure 12).

## 3. Experimental 

### 3.1. Preparing Glyoxlase-1 Enzyme

Glo1 enzyme was downloaded from Protein Data Bank (PDB code: 1QIN) [18]. The *prepare protein* protocol available in DS 3.1 was employed to prepare the enzyme for further processing; this protocol standardizes atoms names, inserts missing loops, calculates pKa and protonates the enzyme. Default parameters were used. 

### 3.2. Creation of Structure-Based Pharmacophore

*Receptor-Ligand Pharmacophore Generation* protocol available in DS 3.1 was used to investigate the essential amino acids that participate in the binding of *S*-(*N*-hydroxy-*N*-*p*-iodophenylcarbamoyl) glutathione (HIPC-GSH) with the enzyme (1QIN). This protocol acts by generating selective pharmacophore models by employing Genetic Function Approximation (GFA) technique. The resulting pharmacophore model is based on receptor-ligand interactions and adds certain features such as hydrogen bond acceptor (HBA), hydrogen bond donor (HBD), hydrophobic (HY), negative ionizable (NI), positive ionizable (PI) and ring aromatic (RA). The essential amino acids will be identified in which the ligand interacts with and these amino acids will be considered later when the final pharmacophore model being built using *Interaction Generation* protocol. This protocol extracts pharmacophore query from the Ludi interaction map which is created inside the active site sphere and only assigns three main features namely HBA, HBD and HY. All the parameters within this protocol were left as their default values [37].

### 3.3. Addition of “Zinc Binding Feature” to the Structure-Based Pharmacophore

As Glo-1 enzyme contains a zinc atom within the active site and inhibitor binding to this atom is indispensible, a customization step is performed to modify the pharmacophore model by adding a zinc binder feature, because this feature is not provided by the *Interaction Generation* protocol. The available protocols in DS 3.1 treat zinc atom as a HBD feature; consequently, there will be no selectivity and preference for the functional groups that specifically bind to zinc atom such as thiols, hydroxamates, and imidazoles. Based on this fact, the HBD feature that orients to the zinc atom has been replaced by zinc feature using *Add Feature From Dictionary* under *Customize Pharmacophore Features* DS 3.1 tools. The zinc feature in the present study was modified to detect the following functional groups (carboxyl, phosphate, imidazole, 1,2,3-triazole, 1,2,4-triazole, tetrazole, thiadiazole, 1-hydroxy-2-oxo derivatives) and we intentionally removed other functional groups although they are considered famous for zinc binding such as (hydroxamates and thiols). Hydroxamates were reported to suffer from poor *in vivo* absorption and liable for metabolism such as glucourination and sulfonation [23,38,39]. Furthermore, hydroxamates could be metabolized to produce hydroxylamines which have potential mutagenicity [40]. For thiols, they have been reported as causing taste disturbances in addition to some cases of photosensitivity in some patients [41].

### 3.4. 2D similarity Search

According to the literature, five compounds (Figure 4) were chosen to be reference structures for extracting similar molecules from a commercial database. The ZINC Clean Drug-like Database which contains 9,542,593 molecules was used for 2D similarity employed to discover similar molecules. To boost our 2D search, three similarity measures search [42,43].

*Find Similar Molecules By Fingerprints* protocols were conducted, namely, *Tanimoto*: *SA/(SA+SB+SC)*, the number of “and” bits normalized by the number of “or” bits, *Dice*: 2*SA/(2*SA+SB+SC), the number of “and” bits normalized by the arithmetic mean of the number of on bits in the target and reference and *Cosine*: SA/((SA+SB)*(SA+SC))^0.5, the number of “and” bits normalized by the geometric mean of the number of on bits in the target and reference. The abbreviations in the equations defined as; *SA*: Bits present in both the target and the reference, *SB*: The number of bits in the target but not the reference and *SC*: The number of bits in the reference but not the target. Within each measure, two properties for similarity check were selected; FCFP_6 and ECFC_6. FCFP_6 is the fingerprint which represents the atom abstraction method used to assign the initial category for atom classes of queried compounds; F stands for “Functional class” which could be HBD, HBA, RA, positive and negative ionizable; C represents the type of the fingerprint to generate and refers to“Extended-Connectivity”, the third and fourth letter are **F** and **P**, which refer to “**F**inger**P**rint”; **P** returns a list of the features present in a molecule with duplications removed; the number after the underscore represents the maximum distance in generating the fingerprint. On the other hand, for ECFC_6, the E is “Atom type” which represents charge and hybridization; the first C “Extended-Connectivity”; and the last C refers to “Counts” which returns to a list of features present in the molecule with duplicates returned [30]. (Figure 7) represents the methodology applied to find similar molecules; within each step, 500 molecules are extracted to be the most similar to each of the target molecules with a total of 15,000 molecules. The resulting molecules were then subjected to further filtration steps such as removing duplicates, selecting drug like compounds according to Lipiniski and Veber rules by applying *Filter by Lipinski and Veber Rules* protocol, Absorption, Distribution, Metabolism, Excretion and Toxicity (ADMET) filtration according to solubility, intestinal absorption and BBB penetration, and then the resulting molecules will be prepared for pharmacophore mapping and docking by applying *Prepare Ligands* protocol which performs ionization, tatumerization and isomerization steps of the resulting molecules [34,35,36]. All the parameters used for the previous protocols were left as default, only the protocol *Filter by Lipinski and Veber Rules* allows one violation of these rules to be tolerated, however, in our work we used strict selection and no violation was allowed by changing the number of violation allowed to zero instead of one.

### 3.5. Mapping 2D Similarity Search Compounds over Structure-Based Pharmacophore

Further selection parameters for the candidate compounds which could be potential inhibitors of Glo-1 are performed by subjecting the resulting compounds from 2D similarity step to be mapped over the structure-based pharmacophore model of Glo-1 enzyme. This step will add further credibility to the selection criteria where the candidate compound should satisfy both 2D similarity of known inhibitors and maps well over the active site of the enzyme. *Screen Library* protocol provided by DS 3.1 was exploited in this step in which default parameters were left unchanged unless mentioned later; this protocol has an advantage of enumerating many subset pharmacophores from a query and screens the input ligands against each pharmacophore. Moreover, it enables pharmacophore features derived from the query to be considered every time the protocol tries to find suitable pharmacophore from the query. Within this work, zinc binder and hydrophobic features were required to be considered every time 2D compounds screened with four minimum features for the numerated pharmacophore. 

### 3.6. Molecular Docking

To increase the reliability and accuracy of Glo-1 inhibitors selection, the extracted compounds from 2D similarity search were further docked on the enzyme (PDB: 1QIN). Three docking programs have been employed. GOLD 5.1 (Genetic Optimization for Ligand Docking; Cambridge Crystallographic Data Centre) [44,45] uses a genetic algorithm to explore the conformational space of the ligands in addition to some flexibility of active site residues. The active site was assigned by referring to a zinc atom with a radius 11Å to cover all the amino acids that are of paramount importance to ligand bonding. GOLD parameters were set to give maximum flexibility to the active site and the ligands in addition to using four scoring functions namely, Goldscore, Chemscore, ASP and CHEMPLP trying to reach optimal accuracy for candidate compound selection, other parameters are not recommended to be changed as this could affect the results. GOLD handles zinc atom by allowing the coordination geometry number to be 4–6 which correlate to tetrahedral, trigonal bipyramidal and octahedral geometries respectively. CDOCKER; provided by DS 3.1 uses a CHARMm-based molecular dynamics (MD) scheme to dock ligands into a receptor binding site; then random conformation will be produced using high temperature molecular dynamics. Once these conformations are translated to the active site, candidate poses are then created using random rigid-body rotations followed by simulated annealing [46]. LibDock, the third docking program used in this study provided by DS 3.1 is a high-throughput algorithm where the ligand will be aligned in the active site based on polar and apolar interaction sites termed as “Hotspots”. For more accurate docking, a CHARMm minimization step was activated [47,48,49], both CDOCKER and Libdock parameters were left unchanged as this could affect the results of the docking. A final step was performed by applying “consensus scoring” tactic, which is based on summing all the docking score from the three docking programs and then the best compounds with the highest scores will be chosen as candidate Glo-1 inhibitors.

## 4. Conclusions 

The present work was intended to discover novel inhibitors of Glo-1 enzyme that selectively bind to the zinc atom in the active site. Understanding the key structural features for Glo-1 inhibition has been achieved by applying structure-based drug design, ligand-based 2D similarity search and molecular docking techniques. The use of an X-ray crystal structure of Glo-1 enzyme has revealed a pharmacophore model indicating the essential features for inhibitor binding especially the presence of zinc atom. The generated pharmacophore contains seven features; a customized ZB, 2HY, 2HBD, HBA and NI. 

Ligand-based 2D similarity search has been conducted by finding five ligands that are active inhibitors for the Glo-1 enzyme based on the literature, and then a comprehensive search for similar ligands from a commercial database called Zinc Clean Drug-Like Database, containing 9,542,593 compounds, has been performed employing diverse similarity search methodologies. The selected compounds were filtered by applying Lipinisks’ and Verbers’ rules in addition to ADMET filters. The resultant candidates were further subjected to molecular docking techniques with consensus scoring to prioritize them. Finally, nine compounds were selected to be candidate inhibitors of Glo-1 enzyme based on the previous techniques in addition to visual investigation of the binding pattern. The nine compounds essentially contain a “zinc binding group” which is considered as an essential functional group for selective Glo-1 inhibitor. The obtained potential inhibitors will be purchased and tested *in vitro* against the enzyme and different cancer cell lines.

## Figures and Tables

**Table 1:** The generated pharmacophore models with their selectivity scores based on Glo-1 enzyme bound to its ligand.

Pharmacophore Summary			
Pharmacophore	Number of Features	Feature Set	Selectivity Score
**Pharmacophore_01**	6	DDDHNN ^a^	8.5416
**Pharmacophore_02**	6	ADDHNN	8.0771
**Pharmacophore_03**	6	ADDHNN	8.0771
**Pharmacophore_04**	6	DDHHNN	7.9040
**Pharmacophore_05**	6	DDDHNN	7.9040
**Pharmacophore_06**	6	ADDHNN	7.7474
**Pharmacophore_07**	6	ADDHNN	7.6186
**Pharmacophore_08**	6	ADDHNN	7.6186
**Pharmacophore_09**	6	ADDHNN	7.5951
**Pharmacophore_10**	6	ADDHNN	7.5951

^a^ D = Hydrogen bond Donor; H = Hydrophobic; N = Negative ionizable; A = Acceptor.

**Table 2:** Structure, consensus scoring and zinc binding groups of candidate compounds as Glo-1 inhibitors.

Name	Structure	Consensus score	ZB group
**Zinc02120846**	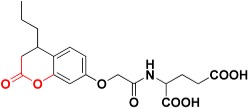	**492.4**	**2H-pyran-2-one**
**Zinc05528245**	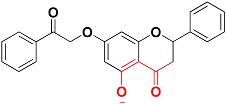	**486.9**	**4-oxo-4H-chromen-5-olate**
**Zinc13100715**	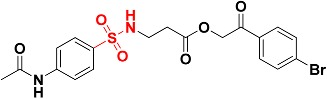	**485.8**	**Sulfonamide**
**Zinc01747330**	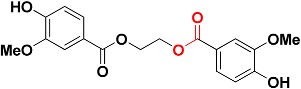	**482.6**	**Ester**
**Zinc06403375**	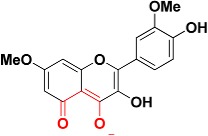	**467.2**	**5-oxo-5H-chromen-4-olate**
**Zinc08791976**	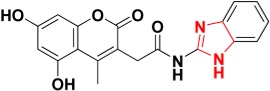	**465.9**	**Imidazole ring**
**Zinc05732763**	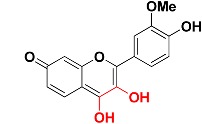	**463.9**	**3,4-dihydroxy-7H-chromen-7-one**
**Zinc00044208**	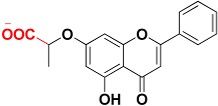	**463.3**	**Carboxylic acid**
**Zinc01863036**	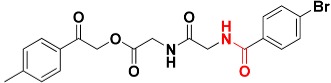	**463.1**	**Amide**
